# The LIM Protein AJUBA is a Potential Oncogenic Target and Prognostic Marker in Human Cancer *via* Pan-Cancer Analysis

**DOI:** 10.3389/fcell.2022.921897

**Published:** 2022-07-11

**Authors:** Na Song, Jia Liu, Ke Zhang, Jie Yang, Kai Cui, Zhuang Miao, Feiyue Zhao, Hongjing Meng, Lu Chen, Chong Chen, Yushan Li, Minglong Shao, Wei Su, Haijun Wang

**Affiliations:** ^1^ School of Basic Medical Sciences, Xinxiang Medical University, Xinxiang, China; ^2^ Key Laboratory of Clinical Molecular Pathology, Department of Pathology, The First Affiliated Hospital of Xinxiang Medical University, Xinxiang, China; ^3^ School of Public Health, Xinxiang Medical University, Xinxiang, China; ^4^ The Second Affiliated Hospital of Xinxiang Medical University, Xinxiang, China

**Keywords:** AJUBA, oncogenic gene, prognostic marker, human cancer, pan-cancer analysis

## Abstract

**Purpose** **:** The LIM (Lin-11, Isl1, MEC-3) domain protein AJUBA is involved in multiple biological functions, and its aberrant expression is related to the occurrence and progression of various cancers. However, there are no analytical studies on AJUBA in pan-cancer.

**Methods:** We performed a comprehensive pan-cancer analysis and explored the potential oncogenic roles of AJUBA, including gene expression, genetic mutation, protein phosphorylation, clinical diagnostic biomarker, prognosis, and AJUBA-related immune infiltration based on The Cancer Genome Atlas and Genotype-Tissue Expression databases.

**Results:** The results revealed that the expression of AJUBA highly correlated with poor clinical outcomes in patients with different types of cancer. Meanwhile, AJUBA expression was positively correlated with cancer-associated fibroblasts in many human cancers, such as breast invasive carcinoma, colon adenocarcinoma, brain lower-grade glioma, lung adenocarcinoma (LUAD), and ovarian serous cystadenocarcinoma (OV). Gene ontology and Kyoto Encyclopedia of Genes and Genomes enrichment analyses showed that AJUBA is mainly involved in protein serine/threonine kinase activity, cell–cell junction, covalent chromatin modification, and Hippo signaling pathway.

**Conclusion:** The pan-cancer study reveals the oncogenic roles of AJUBA and provides a comprehensive understanding of the molecular biological genetic information of AJUBA in various tumors.

## Introduction

Cancer has become the leading cause of mortality worldwide, especially in China. Studies have reported that there were nearly 19.3 million new cancer cases and 10 million cancer deaths worldwide in 2020 (Sung et al., 2021). Carcinogenesis is a multi-step, multi-gene, and multi-stage complex process involving the oncogene activation and inactivation of tumor suppressor genes (Wang H. et al., 2020). Thus, it is essential to conduct a pan-cancer analysis of target genes and reveal its relationship of prognostic value and the particular molecular mechanisms in view of complex tumorigenesis. Hence, we use the public database The Cancer Genome Atlas (TCGA) and Genotype-Tissue Expression (GTEx) project, which contain different functional cancer genomics datasets, to perform the pan-cancer analysis (Cancer Genome Atlas Research Network et al., 2013; Akbani et al., 2014; Keen and Moore, 2015).

The Lin-11, Isl1, and MEC-3 (LIM) protein AJUBA, also known as JUB, contains three tandem LIM regions at the C-terminus and the proline-rich N-terminal preLIM domain (Schimizzi and Longmore, 2015). The AJUBA protein shuttles between the cytoplasm and the nucleus, which depend on the nuclear exporting sequence in the preLIM domain and the nuclear localization sequence in the LIM domain (Jia et al., 2020). The LIM protein AJUBA is a scaffold or adapter protein that participates in various cellular processes such as cell–cell adhesion (Razzell et al., 2018; Rauskolb et al., 2019), gene transcription, mitosis (Hirota et al., 2003; Ferrand et al., 2009), cytoskeletal organization (Rauskolb et al., 2014; Razzell et al., 2018), and cell differentiation, proliferation, and migration (Dommann et al., 2020). Furthermore, studies have established a functional link between AJUBA and tumorigenesis, such as in colorectal cancer (Jia H. et al., 2017), hepatocellular carcinoma (Liu et al., 2018), pancreatic cancer (Zhang et al., 2019), esophageal squamous cell carcinoma (ESCC) (Shi et al., 2016), head and neck squamous cell carcinoma (HNSC) (Loganathan et al., 2020), gastric cancer (Li et al., 2019), prostate cancer (Jia L. et al., 2017), breast cancer (Xu et al., 2019), intrahepatic cholangiocarcinoma (Li et al., 2020), and glioma (Zhang et al., 2021). The high expression of AJUBA is related to tumor epithelial–mesenchymal transition (EMT), migration, and metastasis and patient survival (Liang et al., 2014; Dommann et al., 2020). However, the potential functions and mechanisms for AJUBA in pan-cancer are still unknown.

In view of the prominent role of AJUBA in various tumors, we performed a pan-cancer analysis of AJUBA expression with the patients’ survival prognosis through TCGA database, Gene Expression Profiling Interactive Analysis (GEPIA), and the human protein atlas (HPA). Furthermore, we analyzed the AJUBA expression, genetic mutation, protein phosphorylation, and immune infiltration *via* cBioPortal, the University of ALabama at Birmingham CANcer data analysis Portal (UALCAN), and Tumor Immune Estimation Resource (TIMER) in a variety of tumors. Subsequently, we also conducted a series of analyses including co-expression analysis, functional enrichment analysis, and functional annotation of AJUBA in liver hepatocellular carcinoma (LIHC), esophageal squamous cell carcinoma (ESCC), and prostate adenocarcinoma (PRAD).

## Materials and Methods

### Gene Expression Analysis

The TCGA database (http://gdc-portal.nci.nih.gov/) and GTEx database (https://gtexportal.org/) were used to investigate the expression of AJUBA in pan-cancers and normal samples. Among them, 33 cancers were comprised in the TCGA and GTEx databases. In addition to, we also analyzed the expression of AJUBA in colon adenocarcinoma (COAD), ESCA, LIHC, and stomach adenocarcinoma (STAD) with violin plots (abbreviation referring to Supplementary Table S1). Herein, the results of gene expression analysis were further validated by the use of TIMER (https://cistrome.shinyapps.io/timer/). Because there is no normal sample in certain tumors, the data were supplemented by TCGA database and GTEx databases with a dot plot. Moreover, we analyzed the AJUBA expression at different clinical and pathological stages (stages I, II, III, and IV) in diverse cancers through the “Stage Plot of Expression Analysis” module from GEPIA (http://gepia2.cancer-pku.cn/). In our study, gene expression levels were shown using a log2 (TPM + 1) scale, where TPM stands for transcripts per million. Meanwhile, we input AJUBA in the “Clinical Proteomic Tumor Analysis Consortium (CPTAC),” and phosphoprotein analysis was performed using the UALCAN database (http://ualcan.path.uab.edu/). The protein expression for breast cancer, ovarian cancer, colon cancer, lung adenocarcinoma (LUAD), clear cell renal cell carcinoma (RCC), and uterine corpus endometrial carcinoma (UCEC) is available with phosphorylation at the S79, S119, S137, S230, Y231, S237, S263, and Y267 sites.

### Immunohistochemical Analysis

The HPA (https://www.proteinatlas.org/) contains an atlas of human protein expression models in normal and tumor cells, tissues, and organs. We used the HPA to recognize the pattern of special protein expression that are differentially expressed in specific tumors. Herein, the expression pattern of AJUBA in normal and tumor tissues [colon and COAD, breast and breast invasive carcinoma (BRCA), lung and LUAD, liver and LIHC] was accomplished by using immunohistochemistry images (Supplementary Table S2).

### Prognosis Survival Analysis

The prognostic survival value of AJUBA in different tumors [lower-grade glioma (LGG), LIHC, sarcoma (SARC), kidney renal clear cell carcinoma (KIRC), skin cutaneous melanoma (SKCM), and adrenocortical carcinoma (ACC)] was analyzed using GEPIA2 (http://gepia.cancer-pku.cn/). In GEPIA2, TCGA tumor patients were divided into the high- and low-expression cohorts based on the high and low cut-off values (50% and 50%), respectively, to obtain the overall survival (OS) and disease-free survival (DFS) analyzed using the log-rank test.

### Clinical Diagnostic Value Analysis

The receiver operating characteristic (ROC) curves were prepared depending on TCGA database (https://portal.gdc.cancer.gov/) to assess the diagnostic performance of AJUBA. From the ROC curves, the area under the ROC curve (AUC) values were calculated using the vertical coordinate of false-positive rate and the horizontal coordinate of true-positive rate among different tumors [PRAD, colon and rectal adenocarcinomas (COADREAD), STAD, cholangiocarcinoma (CHOL), COAD, ESCA, HNSC, kidney chromophobe (KICH), rectum adenocarcinoma (READ), glioblastoma multiforme (GBM), acute myeloid leukemia (LAML), pancreatic adenocarcinoma (PAAD), SKCM, thymoma (THYM), oral squamous cell carcinoma (OSCC), and LGG).

### Genetic Alteration Analysis

cBioPortal (http://www.cbioportal.org/) is a powerful website for gene analysis, providing visualization tools for research and analysis of cancer genetic data (Gao et al., 2013). We input AJUBA in the “Quick Search Beta!” module to obtain the characteristic mutations of AJUBA in TCGA tumors. The outcomes of alteration frequency, type of mutation, and copy number alteration of the AJUBA gene were processed using the “Cancer Types Summary” in the cBioPortal database. Meanwhile, the information of mutation sites of AJUBA can be manifested using the protein structure graph or the 3D structural maps through the “Mutations” section. Furthermore, AJUBA gene alteration was associated with clinical prognosis [progression-free survival (PFS), disease-specific survival (DSS), DFS, and OS] in UCEC, which were obtained in the “Comparison” module. The survival curves were shown using the log-rank *p*-value.

### Immune Infiltration Analysis

TIMER is an online open website resource to evaluate the correlation of AJUBA expression with immune infiltration level across diverse TCGA cancers (http://timer.cistrome.org/). In the “Immune-Gene” module, the results of immune infiltration were calculated automatically using the TIMER, EPIC, MCPCOUNTER, XCELL, and TIDE algorithms. The purity-adjusted Spearman’s rho across several cancer types was displayed using the heatmap with numbers. In addition, the scatterplot was popped out to present the relationship between immune infiltration in different cancers of the TCGA database *via* clicking the heatmap in the “Gene-Correlation” module of TIMER2. The correlations between AJUBA expression and immune infiltration were calculated using the purity-adjusted partial Spearman’s rank correlation test.

### Gene Enrichment Analysis

The gene expression data of LIHC, ESCA, and PAAD in HTSeq-FPKM were downloaded from the TCGA database (https://portal.gdc.cancer.gov/) for analysis. In this study, the number of samples in LIHC, ESCA, and PAAD was 424, 173, and 182, respectively. In addition, the co-expressed genes of AJUBA in LIHC, ESCA, and PAAD were screened using Pearson correlation coefficients (*r* > 0.4 or *r* < −0.3 and *p* < 0.001). Meanwhile, we performed an intersection analysis using a Venn diagram to estimate the genes that interact and bind with AJUBA. Gene ontology (GO) and Kyoto encyclopedia of genes and genome (KEGG) enrichment analyses were achieved using co-expressed genes to investigate probable biological functions and signaling pathways affected by AJUBA in LIHC, ESCA, and PAAD. Gene set enrichment analysis (GSEA) is a knowledge-based method for interpreting genome-wide expression profiles (Subramanian et al., 2005). GSEA was performed *via* MSigDB collections (https://www.gsea-msigdb.org/gsea/ msigdb/index.jsp). The enrichment analysis of AJUBA was visualized using the “clusterProfiler” and “ggplot2” R packages (Yu et al., 2012). Statically significant difference was regarded when *p*-value < 0.05.

### Statistical Analyses

Data are presented as means ± standard error (SD). Differences between groups were analyzed using a Student’s *t*-test. Genes correlation analysis was performed using Spearman correlation coefficients. Statistical analyses were performed using R 3.6.3. *p* < 0.05 (two-tailed) was considered statistically significant (*p < 0.05, **p < 0.01, ***p < 0.001).

## Results

### Gene Expression Analysis of AJUBA

**FIGURE 1:**
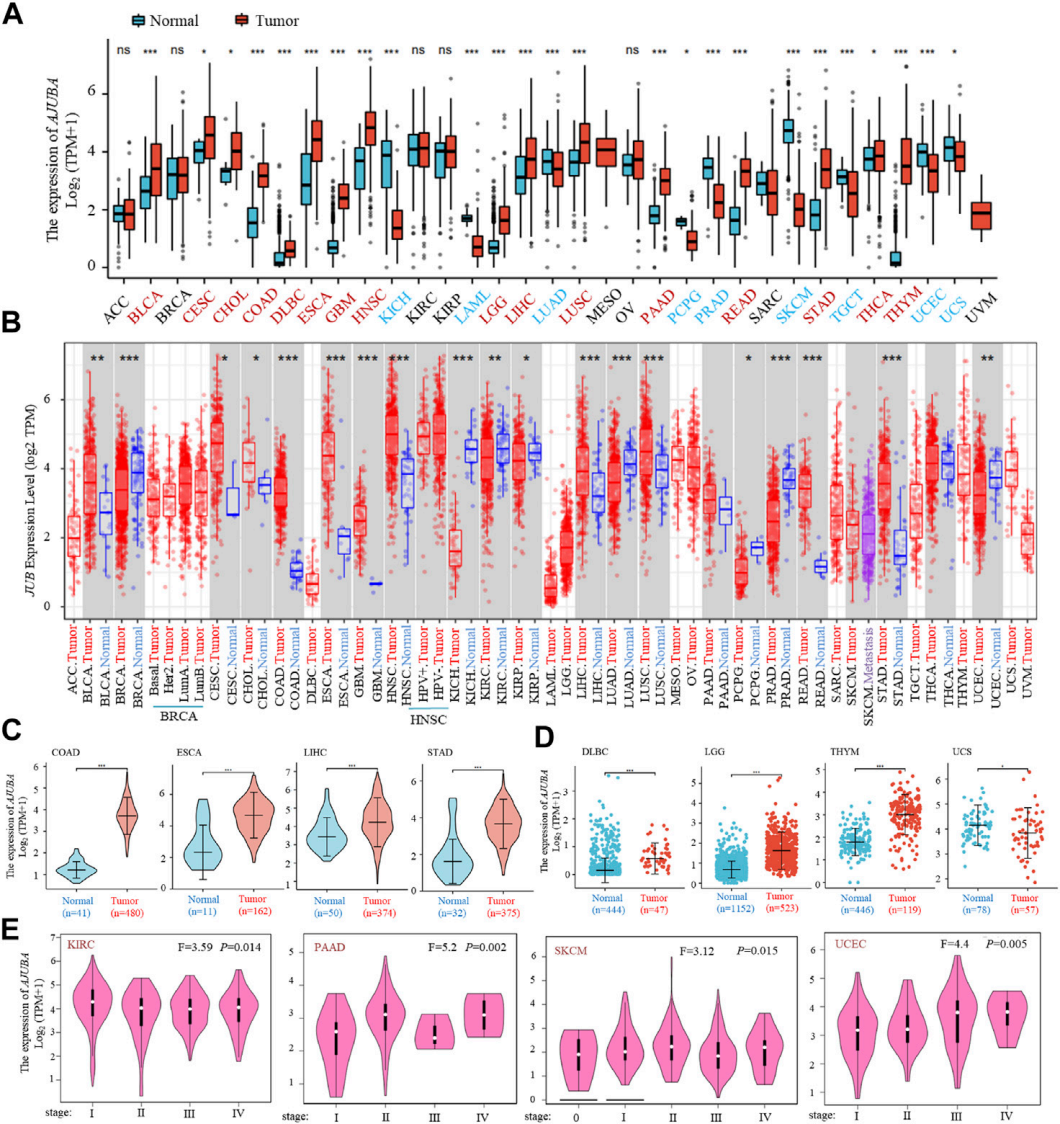
AJUBA gene expression in different cancers and pathological stages. **(A)** gene expression profiles of the AJUBA gene in various cancers was analyzed *via* The Cancer Genome Atlas (TCGA) and Genotype-Tissue Expression (GTEx) databases. **(B)** the expression status of the AJUBA gene in various cancers or specific subtypes of cancer was performed using Tumor Immune Estimation Resource 2 (TIMER2). **(C)** we analyzed the expression of AJUBA in colon adenocarcinoma (COAD), esophageal squamous cell carcinoma (ESCA), liver hepatocellular carcinoma (LIHC), and stomach adenocarcinoma (STAD) with violin plots separately as a supplementary note on **(A)**. **(D)** using the dot plot data to analyze the type of diffuse large B-cell lymphoma (DLBC), lower-grade glioma (LGG), thymoma (THYM), and uterine carcinosarcoma (UCS) in TCGA, for which the corresponding normal tissues in the GTEx database were contained as controls. **(E)** AJUBA gene expression was analyzed using different pathological stages (stage 0, stage I, stage II, stage III, and stage IV) of kidney renal clear cell carcinoma (KIRC), pancreatic adenocarcinoma (PAAD), skin cutaneous melanoma (SKCM), and uterine corpus endometrial carcinoma (UCEC) *via* TCGA data. Log2 (TPM + 1), where TPM stands for transcripts per million, was utilized for the log scale. *p < 0.05; **p < 0.01; ***p < 0.001.

Initially, AJUBA expression levels in pan-cancer were examined in the TCGA and GTEx databases. The analysis revealed that AJUBA expression was significantly higher in various tumors than in the respective normal tissues, including bladder urothelial carcinoma (BLCA), cervical squamous cell carcinoma and endocervical adenocarcinoma (CESC), CHOL, COAD, lymphoid neoplasm diffuse large B-cell lymphoma (DLBC), ESCA, GBM, HNSC, brain LGG, LIHC, lung squamous cell carcinoma (LUSC), PAAD, READ, and STAD as well as thyroid carcinoma and THYM. In contrast, AJUBA expression was low in KICH, LAML, LUAD, pheochromocytoma and paraganglioma (PCPG), PRAD, SKCM, testicular germ cell tumors (TGCT), UCEC and uterine carcinosarcoma (UCS) (*p* < 0.05) (Figure 1A). In a similar manner, we also assessed expression among various cancer types using the TIMER2 database. As shown in Figure 1B, AJUBA expressions in human tumors of BLCA, CESC, CHOL, COAD, ESCA, GBM, HNSC, LIHC, LUSC, READ, and STAD were dramatically higher than expressions in paired normal groups (*p* < 0.05). In addition, as we know that the increased methylation level leads to the decreased expression of target gene, the AJUBA promoter methylation level was analyzed using the UALCAN database. The results demonstrated that the methylation levels of the AJUBA promoter in most tumors were significantly compared with the normal tissues (Supplementary Figure S1).

Specifically, we further analyzed AJUBA expression in digestive system tumors (COAD, ESCA, LIHC, and STAD) (*p* < 0.001) (Figure 1C). In addition, as shown in Figure 1D, the normal tissue of the GTEx dataset was provided as supplementary to further evaluate the expression difference of AJUBA between the normal groups and cancer groups in DLBC, LGG, THYM, and UCS. We also explored the correlation between AJUBA expression level and the pathological stages of tumors, including in KIRC, PAAD, SKCM and UCEC (*p* < 0.01) using the “Pathological Stage Plot” module of GEPIA2 (Figure 1E).

**FIGURE 2:**
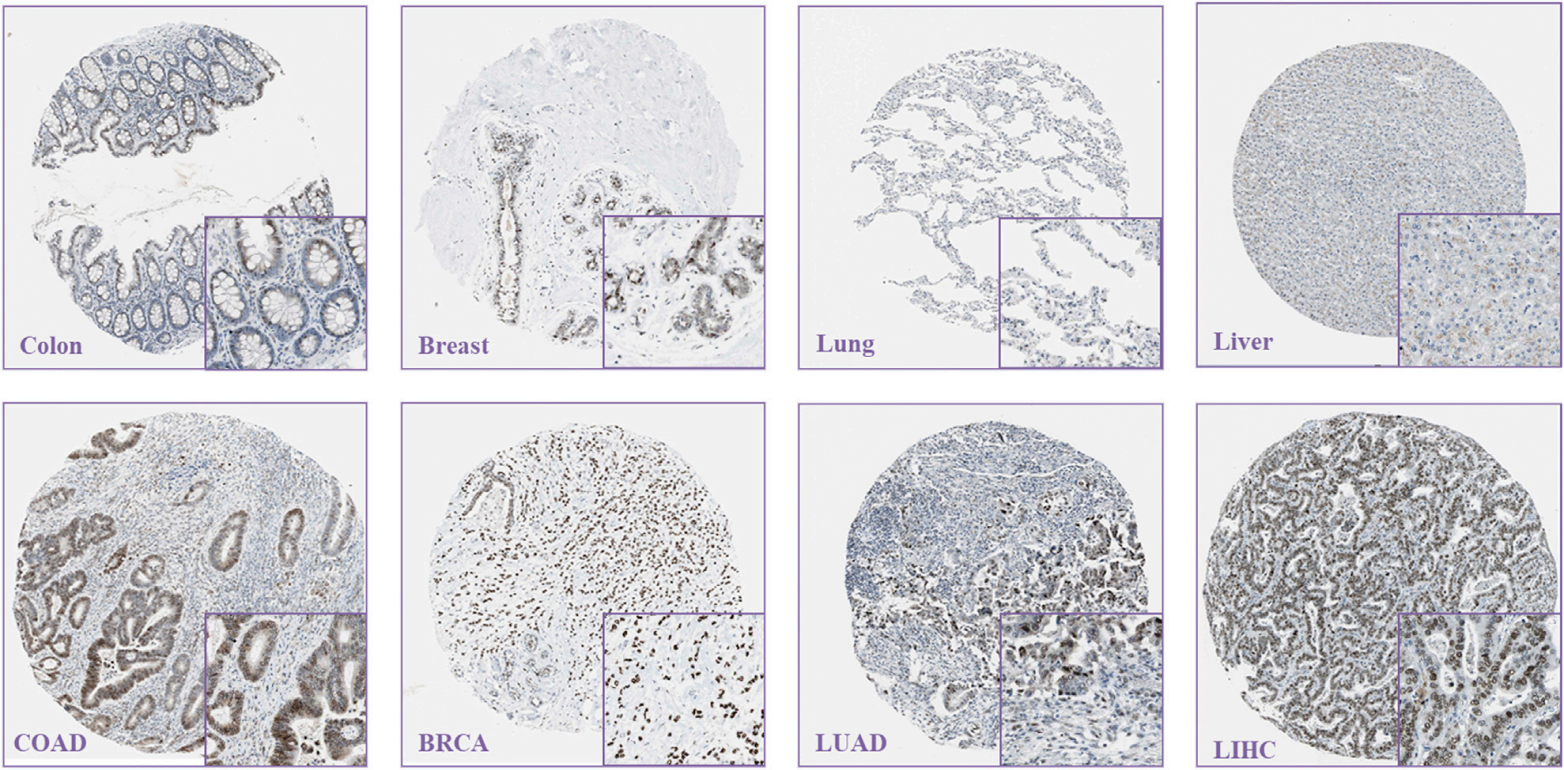
Representative immunohistochemical staining results of AJUBA expression in tumor tissues and their normal tissues via the human protein atlas (https://www.Proteinatlas.org/).

Meanwhile, the HPA database was used to assess the AJUBA expression levels among different tumors. The results displayed that higher AJUBA expression levels were observed in different tumors (COAD, BRCA, LUAD, and LIHC). In contrast, its expression is low in normal tissue (colon, breast, lung, and liver) (Figure 2). These results demonstrated that AJUBA may play a significant role in various cancers.

### Survival Analysis Data of AJUBA

**FIGURE 3:**
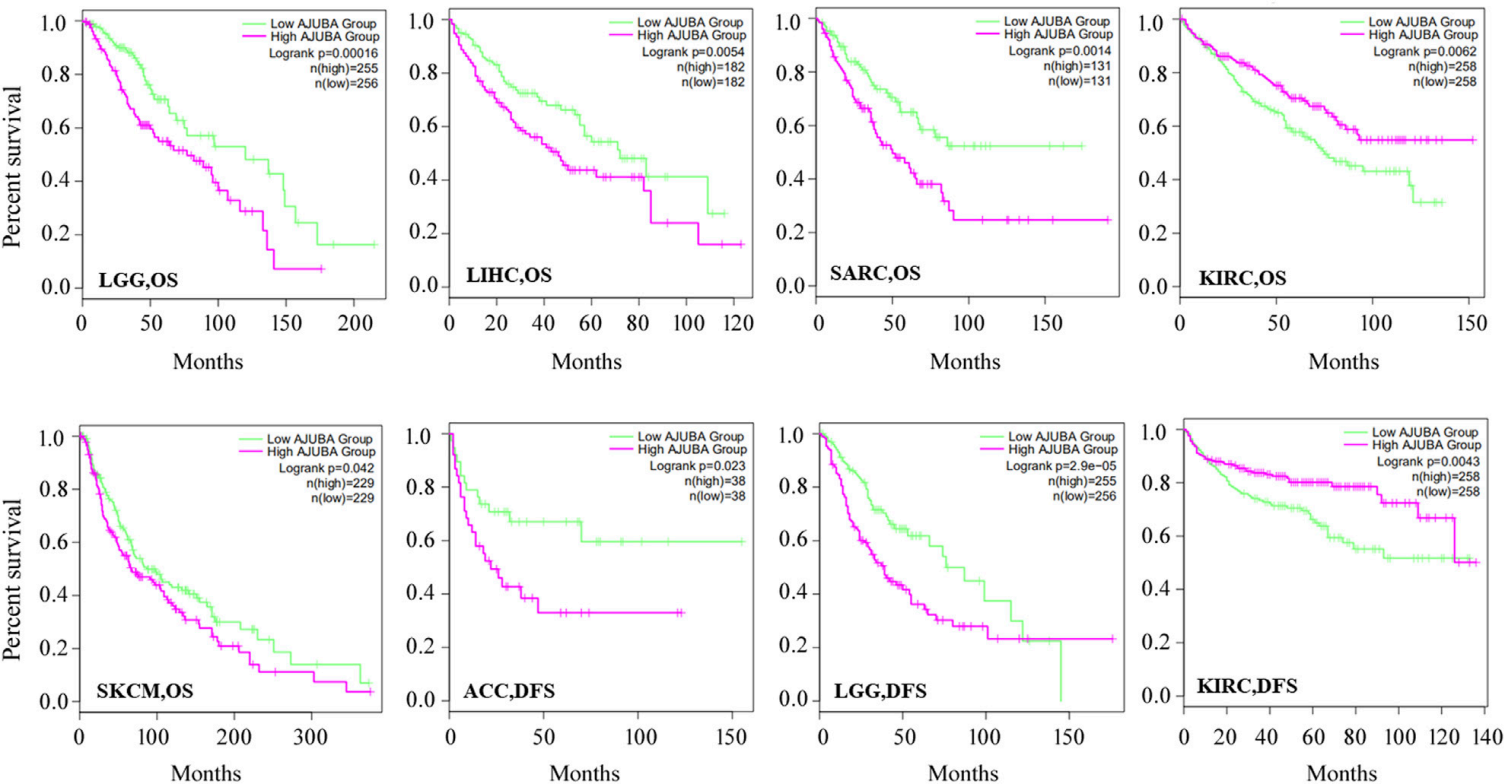
Using the gene expression profiling interactive analysis 2 (GEPIA2) database (http://gepia.cancer-pku.cn/), correlation between the AJUBA gene expression and survival prognosis of LGG, LIHC, sarcoma (SARC), KIRC, SKCM, and adrenocortical carcinoma (ACC) was displayed with the overall survival (OS) or disease-free survival (DFS) curves.

Further, to evaluate the value of AJUBA in clinical prediction, the correlation between AJUBA expression and OS and DFS was analyzed *via* the GEPIA2 database. For OS, higher expression of AJUBA was dramatically associated with decreased OS in LGG (*p* = 0.00016), LIHC (*p* = 0.0054), SARC (*p* = 0.0014), and SKCM (*p* = 0.042). Higher AJUBA expression was significantly associated with reduced DFS in ACC (*p* = 0.023) and LGG (*p* = 2.9e−05). In contrast, it was shown in KIRC that as the expression of AJUBA increases, its OS (*p* = 0.0062) and DFS (*p* = 0.0043) also increase (Figure 3).

### Clinical Diagnostic Value Analysis of AJUBA

**FIGURE 4:**
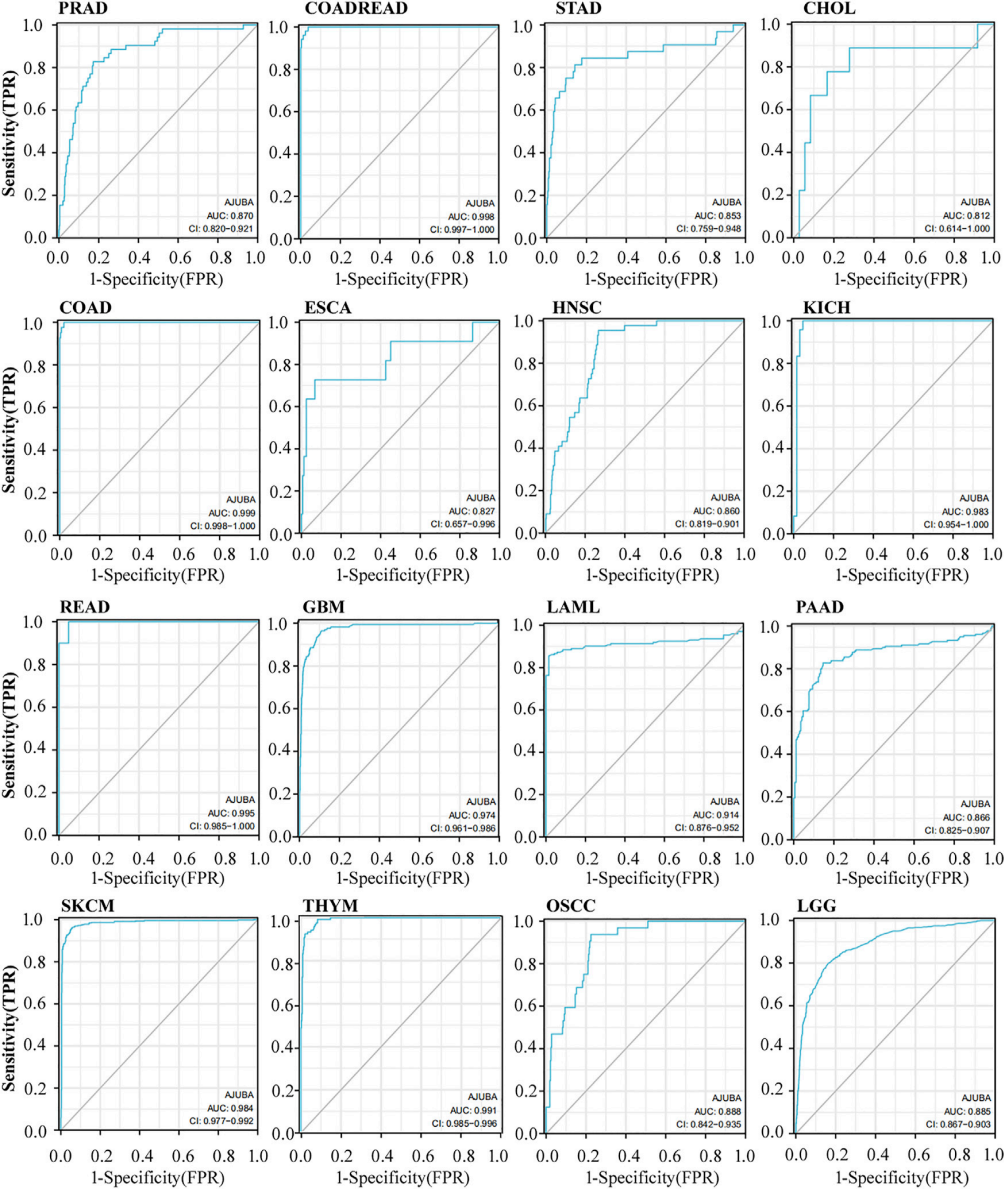
The diagnostic value of AJUBA in different cancers, including prostate adenocarcinoma (PRAD), colon and rectal adenocarcinomas (COADREAD), STAD, CHOL, COAD, esophageal squamous cell carcinoma (ESCA), head and neck squamous cell carcinoma (HNSC), kidney chromophobe (KICH), rectum adenocarcinoma (READ), glioblastoma multiforme (GBM), acute myeloid leukemia (LAML), pancreatic adenocarcinoma (PAAD), SKCM, and thymoma (THYM), as well as oral squamous cell carcinoma (OSCC) and LGG.

ROC curve analysis was used to assess the diagnostic value of AJUBA in various cancers patients. Specifically, the closer the AUC value is to 1, the better the prediction performance. As shown in Figure 4, results reveal the high diagnostic value of AJUBA in OADREAD, COAD, KICH, READ, GBM, LAML, SKCM, and THYM (AUC > 0.9). Moreover, the analysis shows that AJUBA has a definite value for diagnosis among PRAD, STAD, CHOL, ESCA, HNSC, and PAAD, as well as OSCC and LGG (0.8 < AUC < 0.9). Moreover, the Nomogram plot were made for the prognosis predicting of cancer patients at 1, 3, and 5 years in different tumors (Supplementary Figure S2). These results demonstrate that AJUBA could be a potential diagnostic marker in pan-cancer.

### AJUBA Mutation in Various Tumors

**FIGURE 5:**
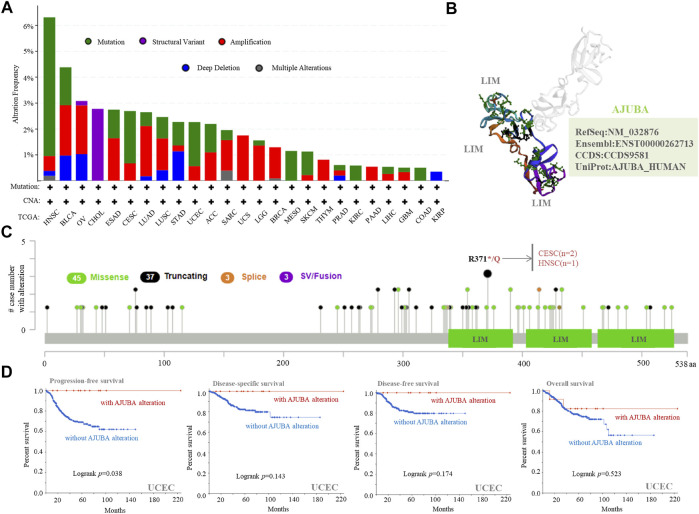
Mutation feature of AJUBA in different cancers of TCGA. The mutation features of AJUBA among the TCGA tumors was analyzed using the cBioPortal tool. The alteration frequency with **(A)** mutation type, **(B)** the 3D structure of AJUBA protein, and **(C)** mutation sites are displayed. **(D)** Using the cBioPortal tool, we analyzed the potential relationship between mutation profiles and progression-free, disease-specific, disease-free, and overall survivals of UCEC.

To investigate the correlation of the AJUBA gene mutation in different cancer types, we analyzed the mutation status of AJUBA through the cBioPortal platform based on TCGA data. AJUBA has the highest mutation frequency in patients with HNSC (6%) in Figure 5A. Strikingly, all patients with CHOL had a structural variant of the AJUBA gene, which displayed an alteration frequency of ∼3%. The 3D structure of the AJUBA protein is presented in Figure 4B. As shown in Figure 4C, we can intuitively understand the types, sites, and case number of the AJUBA genetic alteration, including the missense, truncating, and fusion mutations. In addition to, missense and truncating of AJUBA were the predominant mutation (Figure 5C). Furthermore, we found that R371*/Q alteration in the LIM domain was detected in two cases of CESC and one case of HNSC. In addition to, we analyzed potential links between genetic alteration of AJUBA and the survival prognosis of patients in various cancers. These results indicated that UCEC patients with altered AJUBA displayed better prognosis in PFS (*p* = 0.038), but not in DFS (*p* = 0.143), DFS (*p* = 0.174) and OS (*p* = 0.523), compared with groups without AJUBA alteration (Figure 5D).

### AJUBA Protein Phosphorylation Analysis

**FIGURE 6:**
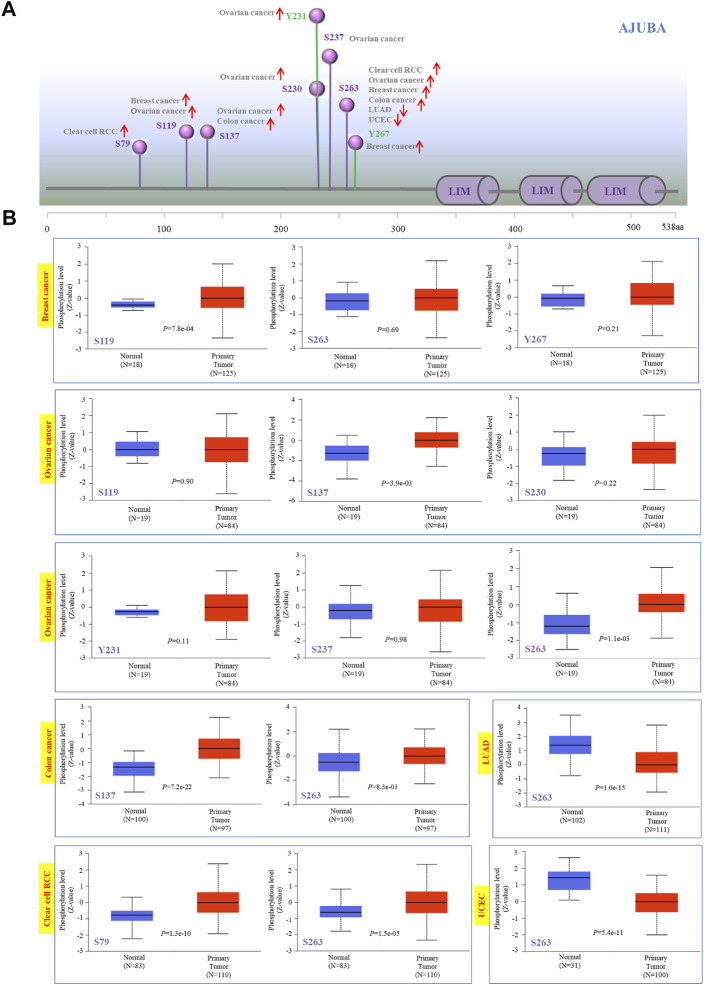
Phosphorylation analysis of AJUBA protein in different cancers. Using the clinical proteomic tumor analysis consortium (CPTAC) dataset, we analyzed the expression level of AJUBA phosphoproteins (S79, S119, S137, S230, Y231, S237, S263, and Y267 sites) between normal tissue and primary tumor tissue through the UALCAN. **(A)** The positive results of phosphoprotein sites are shown in the schematic diagram of the AJUBA protein. **(B)** We also presented the box plots for various cancers, including breast cancer, ovarian cancer, colon cancer, lung adenocarcinoma (LUAD), clear cell renal cell carcinoma (RCC), and UCEC.

We made use of the CPTAC database to explore the phosphorylation of AJUBA between cancer tissues and normal tissues. Figure 6A displays the sites of AJUBA phosphorylation. Meanwhile, we found that three types of tumors (ovarian cancer, colon cancer, and renal clear cell carcinoma) show a higher phosphorylation level in all primary cancer tissues than do normal tissues at the S263 locus of AJUBA (*p* < 0.05), followed by an increased phosphorylation level of the S119 locus for breast cancer (*p* = 7.8e−04) and the S137 locus for ovarian cancer (*p* = 0.0039) and colon cancer (*p* = 7.2e−22) (Figure 6B). In contrast, lower protein phosphorylation of AJUBA was detected in LUAD and UCEC at the S263 locus of AJUBA (*p* < 0.001) (Figure 6B). Therefore, these results reveal at least the importance of the phosphorylation of S263, S119, and S137 in tumor development.

### Immune Infiltration Analysis of AJUBA

**FIGURE 7:**
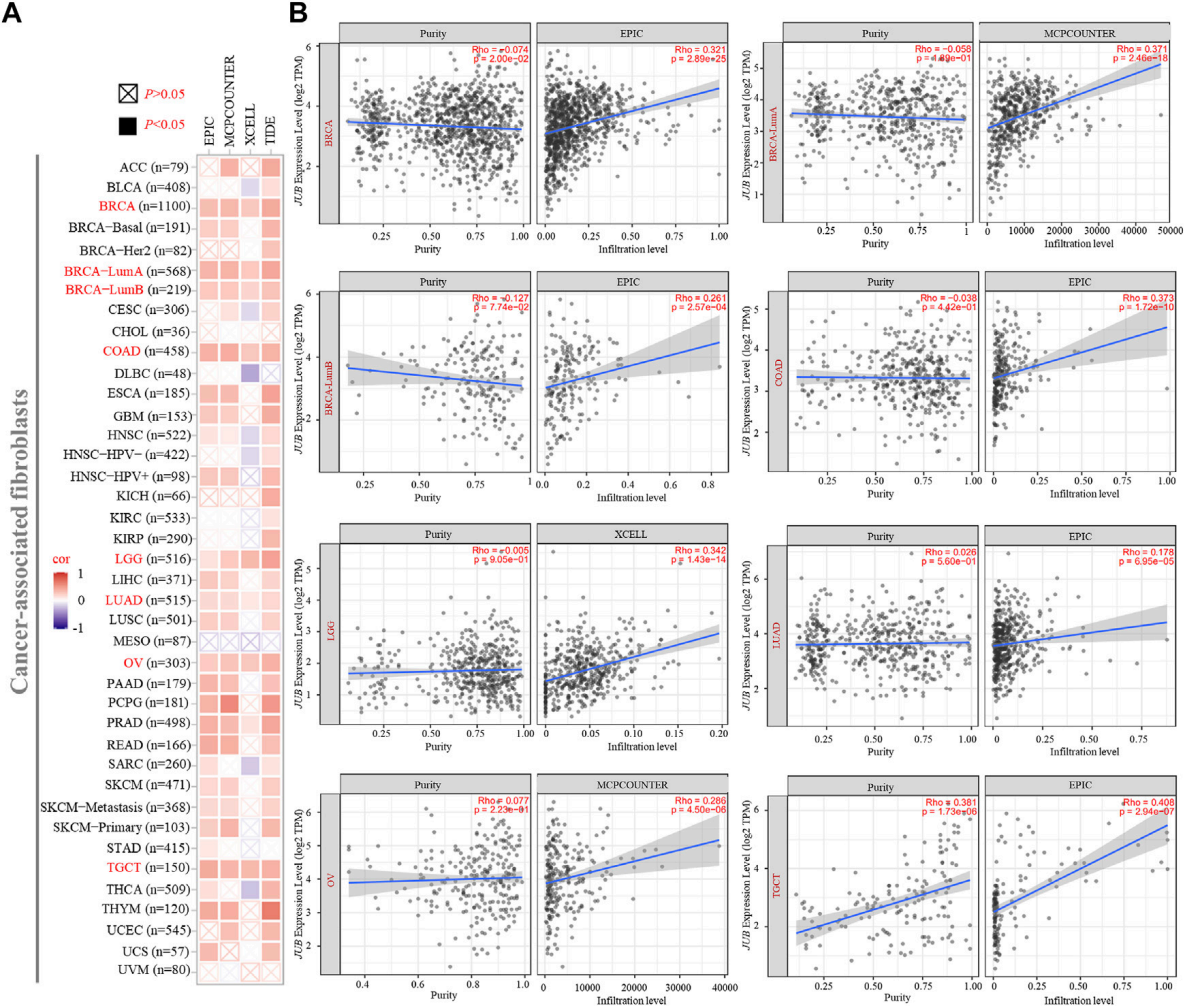
Correlation analysis between the AJUBA gene expression and immune infiltration of cancer-associated fibroblasts (CAFs). Different algorithms analyzed potential correlations in **(A)** the AJUBA expression level and **(B)** infiltration of CAFs among different cancer types based on TCGA database.

Immune infiltration plays a significant role in the process of various tumor initiation, progression, and metastasis. As an important part of the immune microenvironment, cancer-associated fibroblasts (CAFs) have various functions in the occurrence and development of tumors; herein, they hold promise as potential targets for cancer therapy (Liu et al., 2019; Sahai et al., 2020). Here, we used the TIMER, EPIC, MCPCOUNTER, XCELL, and TIDE algorithms to perform a comprehensive exploration of correlation between the infiltration level of CAFs and AJUBA gene expression in a variety of tumor types of TCGA (Figure 7A). Through a series of analyses, a statistical positive correlation was observed between the immune infiltration of CAFs and AJUBA expression in BRCA, BRCA-LumA, BRCA-LumB, COAD, LGG, LUAD, ovarian serous cystadenocarcinoma (OV), and TGCT based on all or most algorithms. As shown in Figure 7B, the scatterplot data of the above cancers were displayed *via* only one algorithm (*p* < 0.001). Furthermore, the correlation between AJUBA and immune checkpoints was also investigated. And the results demonstrated that AJUBA was positively associated with a few immunosuppressive checkpoints in the vast majority of tumors, including PD-L1, PD-L2, PD-1, CTLA4, LAG3, TGFB1/TGFBR1, TIGT, TIM-3, and IL10/IL10RB (Supplementary Figure S3). Above all, these findings strongly suggest that AJUBA promotes tumor development *via* enhancing the level of CAF immune infiltration and being positively associated with immunosuppressive checkpoints.

### Functional Enrichment Analysis of AJUBA

**FIGURE 8:**
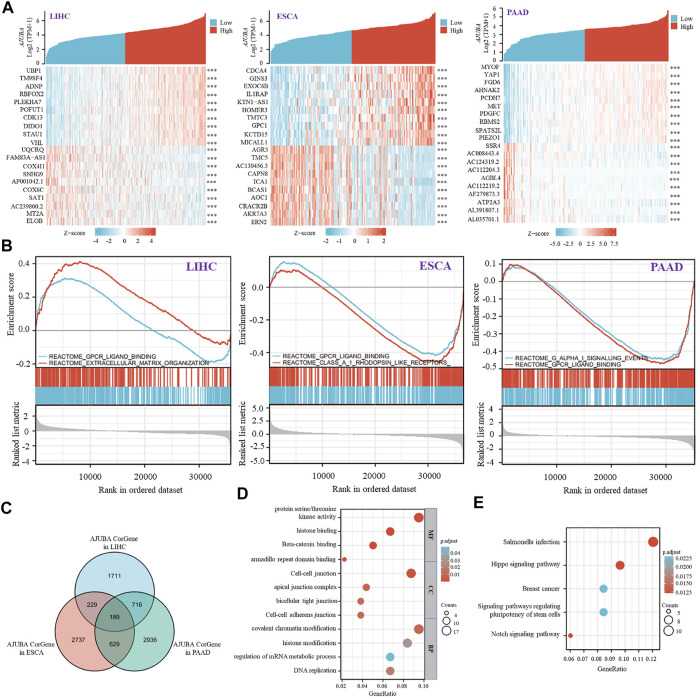
AJUBA-related gene enrichment analysis. **(A)** the heatmap shows the top 10 genes with positive and negative co-expression of AJUBA in LIHC, ESCA, and pancreatic adenocarcinoma (PAAD) *via* TCGA database. **(B)** gene set enrichment analysis (GSEA) was performed in LIHC, ESCA, and PAAD based on MSigDB collections. **(C)** intersection co-expression genes of AJUBA in LIHC, ESCA, and PAAD. Note: *r* > 0.4 or *r* < −0.3 and *p* < 0.001. **(D,E)** based on the interacted genes of AJUBA in **(C)**, gene ontology (GO) analysis and Kyoto Encyclopedia of Genes and Genomes (KEGG) pathway analysis were performed.

To further study the molecular mechanism of the AJUBA during tumor progression, we tried to screen out the AJUBA-related genes *via* a series of functional enrichment analyses. Initially, in the TCGA database, we screened 2,828 positively correlated genes and 17 negatively correlated genes in LIHC, 1,781 positively correlated genes and 2,017 negatively correlated genes in ESCA, and 3,452 positive and 1,018 negative genes in PAAD through Spearman correlation coefficients (*r* > 0.4 or *r* < −0.3 and *p* < 0.001). Moreover, the top 10 co-expressed genes with positive and negative correlation are presented in the form of a heatmap (Figure 8A). Meanwhile, the Venn diagram revealed that 189 genes were discovered to be co-expressed with AJUBA in LIHC, ESCA, and PAAD (Figure 8C). As demonstrated in Figure 8B, GSEA provides insight into novel reactome pathways, which indicated reactome_extracellular_matrix_organization, reactome_class_ a_1_rhodopsin_like_receptors, and reactome_G_alpha_i_signalling_events were separately enriched in LIHC, ESCA, and PAAD. However, reactome_gpcr_ligand_binding was enriched in LIHC, ESCA, and PAAD (Figure 8B). We used the TCGA database to perform GO and KEGG enrichment analyses. The GO data of Figure 8D indicate that “protein serine/threonine kinase activity,” “cell–cell junction,” and “covalent chromatin modification” might play an important role in tumorigenesis and development. As demonstrated in Figure 8B, the KEGG enrichment analysis data further reveal that a number of these genes exhibit a certain level of correlation with Salmonella infection and the Hippo signaling pathway.

## Discussion

It has been reported that the LIM protein AJUBA participates in a series of cell biological processes among different species including cell adhesion, cell mitosis, cell apoptosis, and signal transduction as well as mechanical force and gene transcription regulation (Goyal et al., 1999; Hirota et al., 2003; Ayyanathan et al., 2007; Rauskolb et al., 2014; Jia H. et al., 2017; McCormack et al., 2017). AJUBA, which can sense mechanical forces, is related to poor prognosis and outcomes in patients with cancers (Li et al., 2019; Le et al., 2020). Furthermore, numerous studies have reported that aberrant AJUBA expression is associated with initiation, progression, and metastasis of various human tumors (Zhang et al., 2020). In this study, we comprehensively explored gene expression, survival outcomes, genetic alteration, and immune infiltration of the AJUBA gene in multiple cancers *via* the TCGA, HPA, and CPTAC databases.

Previous studies have shown that the different molecular mechanisms of AJUBA promote tumorigenesis and development in a variety of cancers. For example, the LIM protein AJUBA promotes the growth of colorectal cancer cells *via* suppression of the JAK1/STAT1/IFIT2 network and activates N-cadherin expression through interaction with Twist in colorectal cancer cells (Jia H. et al., 2017; Wu et al., 2021). In hepatocellular carcinoma, AJUBA is activated by TCF4 and participated in EMT (Zhang et al., 2020). Meanwhile, AJUBA promotes the migration and invasion of cells *via* upregulating MMP10 and MMP13 expression (Shi et al., 2016) with poor clinical prognosis (Zhang et al., 2018) in ESCC. In pancreatic cancer, the AJUBA/SP1 complex could motivate SP1 target gene transcription and promote cell proliferation (Zhang et al., 2019). Although a number of studies have indicated that AJUBA plays an oncogene role to promote tumorigenesis, several literatures also demonstrated that AJUBA inhibits cell growth *via* targeting of β-catenin and YAP signaling in hepatocellular carcinoma (Liu et al., 2018), and AJUBA might play a tumor suppressor role in ESCC (Zhang et al., 2015).

According to the TCGA, HPA cohort, and GEPIA datasets, the gene expression analysis demonstrated that aberrant expression of AJUBA occurred usually across a variety of cancer types. We confirmed that AJUBA expression was distinctively upregulated in different tumors compared with counterpart normal tissues, such as BLCA, COAD, DLBC, ESCA, CBM, HNSC, LGG, LIHC, LUSC, PAAD, and READ as well as STAD and THYM. However, AJUBA gene expression is downregulated in KICH, LAML, LUAD, PRAD, SKCM, TGCT, and UCEC. Therefore, differential AJUBA gene expression in different cancers revealed that AJUBA may have diverse biological functions across various cancer types. In addition, abnormal expression levels of AJUBA are correlated with poor prognosis among many human cancer types, which vigorously reveals that AJUBA is a potential prognostic biomarker in cancer patients.

Gene mutations play an essential role in the occurrence and development of tumors (Tamborero et al., 2013; Martinez-Jimenez et al., 2020). In our study, missense and truncating mutations are the most leading DNA alterations of AJUBA gene across TCGA tumors. Meanwhile, special gene mutations to a certain extent may predict the prognosis of patients. Afterwards, we made use of the CPTAC database to analyze the molecular mechanism of the AJUBA protein in breast cancer, ovarian cancer, colon cancer, LUAD, clear cell RCC, and UCEC in the field of total and phosphorylated proteins. This study has indicated that a high expression level of AJUBA total and phosphorylated proteins is found at most loci with the preLIM domain. Notwithstanding, we still found a low expression level of AJUBA and phosphorylated proteins at the S263 locus in LUAD and UCEC. Therefore, additional experiments need to be done in order to clarify the functions of the S263 locus in various cancers and the corresponding mechanisms in tumorigenesis.

The tumor microenvironment (TME) is essential for tumorigenesis and tumor development. Previous studies have indicated that the TME contains various components, such as CAFs, mesenchymal stem cells, endothelial cells, and lymphocytes (Joyce and Fearon, 2015; Ridge et al., 2017). AJUBA plays a vital role in the extracellular matrix (ECM), because of its significant characteristic in terms of focal adhesion (Nola et al., 2011; Razzell et al., 2018; Rauskolb et al., 2019). Moreover, CAFs have an effect on creating ECM structure and tumor immunity functions and promote cancer cell growth, proliferation, and invasion (Kalluri, 2016). Therefore, we used the TIMER, EPIC, MCPCOUNTER, XCELL, and TIDE algorithms to demonstrate the correlation between the AJUBA gene and the CAFs in various cancer types. This relationship between AJUBA expression and CAFs might be another reason for the poor prognosis and metastasis of AJUBA in various type of cancers. Moreover, AJUBA was positively correlated with few of immunosuppressive checkpoints, such as PD-L1, PD-L2, PD-1, CTLA4, LAG3, TGFB1/TGFBR1, TIGIT, TIM-3, and IL10/IL10RB (Fujio et al., 2017; Shi et al., 2018; Ruffo et al., 2019; Dixon et al., 2021; Lentz et al., 2021). These results suggest that AJUBA promotes tumor progression by altering the TME.

Several studies have demonstrated that AJUBA expression has a positive correlation with the EMT process in different cancers, including in colorectal cancer (Liang et al., 2014) and hepatocellular carcinoma (Zhang et al., 2020). On the one hand, some reports found that the EMT boosts the generation of CAFs (Orr et al., 2012); on the other hand, CAFs have been reported to promote EMT (Giannoni et al., 2010; Chen et al., 2014; Yu et al., 2014). Taken together, these results reveal that aberrant AJUBA expression is associated with the TME and EMT to promote angiogenesis, invasion, and metastasis in various cancer types.

GO enrichment analysis and KEGG pathway enrichment analysis were performed to explore the molecular function, biological process, cellular components (CC), and signaling pathway analysis of the AJUBA gene in LIHC, ESCA, and PAAD. In this study, we found that the AJUBA gene is mainly involved in protein serine/threonine kinase activity, cell–cell junction, covalent chromatin modification, and the Hippo signaling pathway. Importantly, the AJUBA gene plays a crucial role in Hippo signaling pathway in many studies. Some studies have investigated that the LIM protein AJUBA is a negative regulator in the Hippo signaling pathway (Das Thakur et al., 2010; Rauskolb et al., 2014). Some works on the mechanism of AJUBA in the Hippo signaling pathway have been conducted, including limiting Hippo regulation by sequestering a cytosolic Hippo kinase complex (Jagannathan et al., 2016) and by EGFR-MAPK signaling (Reddy and Irvine, 2013). In addition to, overexpression of AJUBA promotes tumorigenesis of colorectal cancer by inactivating Hippo signaling (Wang X. et al., 2020). In contrast, AJUBA negatively regulates YAP activity through the LATS family to suppress cell proliferation in malignant mesothelioma (Tanaka et al., 2015). Consequently, AJUBA plays a vital role in the occurrence and development of tumors *via* the Hippo signaling pathway.

In our study, we carried out the pan-cancer analysis of AJUBA in different cancers and investigated the relationship of its aberrant expression with the patient’s prognosis. However, although we used different public platforms, including the TCGA, GTEx, and CPTAC databases, data about some special cancer types were still restricted. In this context, the need for mechanistic studies to reveal the functions of AJUBA at the molecular level is more than urgent.

## Conclusion

Taken together, the results of this pan-cancer analysis revealed that the abnormal expression of AJUBA relates with clinical prognosis, gene mutation, protein phosphorylation, and immune cell infiltration in various cancer types. Furthermore, these studies supplied a comprehensive bioinformatics analysis of AJUBA in pan-cancer tumor types. The results reveal that the AJUBA gene is considered as a potential clinical biomarker for the prognosis of cancer patients, especially in LIHC, ESCA, and PAAD.

## Data Availability

The datasets presented in this study can be found in online repositories. The names of the repository/repositories and accession number(s) can be found in the article/Supplementary Material.

## Supplementary Material

Supplementary Table S2 AJUBA expression sample information based on TCGA and GTEx 691 databases.
