# Supplementary material for: The LIM Protein AJUBA is a Potential Oncogenic Target and Prognostic Marker in Human Cancer via Pan-Cancer Analysis
Source: Front Cell Dev Biol. 2022 Jul 11;10:921897. doi: 10.3389/fcell.2022.921897 (PMC9309301; doi:10.3389/fcell.2022.921897)
Supplement: Supplementary file 2 [file Table1.DOCX]

**Supplementary Table 1** The abbreviation and full name

| **Abbreviation** | **Full Name** |
| --- | --- |
| ACC | Adrenocortical carcinoma |
| BLCA | Bladder Urothelial Carcinoma |
| BRCA | Breast invasive carcinoma |
| CESC | Cervical squamous cell carcinoma and endocervical adenocarcinoma |
| CHOL | Cholangiocarcinoma |
| COAD | Colon adenocarcinoma |
| COADREAD | [Colorectal adenocarcinoma](https://gdac.broadinstitute.org/runs/stddata__latest/samples_report/COADREAD.html) |
| DLBC | Lymphoid Neoplasm Diffuse Large B-cell Lymphoma |
| ESCA | Esophageal carcinoma |
| GBM | Glioblastoma multiforme |
| HNSC | Head and Neck squamous cell carcinoma |
| KICH | Kidney Chromophobe |
| KIRC | Kidney renal clear cell carcinoma |
| KIRP | Kidney renal papillary cell carcinoma |
| LAML | Acute Myeloid Leukemia |
| LGG | Lower Grade Glioma |
| LIHC | Liver hepatocellular carcinoma |
| LUAD | Lung adenocarcinoma |
| LUSC | Lung squamous cell carcinoma |
| MESO | Mesothelioma |
| OV | Ovarian serous cystadenocarcinoma |
| OSCC | Oral squamous cell carcinoma |
| PAAD | Pancreatic adenocarcinoma |
| PCPG | Pheochromocytoma and Paraganglioma |
| PRAD | Prostate adenocarcinoma |
| READ | Rectum adenocarcinoma |
| SARC | Sarcoma |
| SKCM | Skin Cutaneous Melanoma |
| STAD | Stomach adenocarcinoma |
| TGCT | Testicular Germ Cell Tumor |
| THCA | Thyroid carcinoma |
| THYM | Thymoma |
| UCEC | Uterine Corpus Endometrial Carcinoma |
| UCS | Uterine Carcinosarcoma |
| UVM | Uveal Melanoma |
| TCGA | The Cancer Genome Atlas |
| GTEx | Genotype-Tissue Expression |
| CAFs | cancer-associated fibroblasts |
| GO | Gene Ontology |
| KEGG | Kyoto Encyclopedia of Genes and Genomes |
| NES | nuclear exporting sequence |
| NLS | nuclear localization sequence |
| EMT | Epithelial-mesenchymal transition |
| GEPIA | Gene Expression Profiling Interactive Analysis |
| HPA | Human Protein Atlas |
| UALCAN | The University of ALabama at Birmingham CANcer data analysis Portal |
| TIMER | the Tumor Immune Estimation Resource |
| TPM | transcripts per million |
| CPTAC | Clinical Proteomic Tumor Analysis Consortium |
| OS | Overall survival |
| DFS | Disease-free survival |
| ROC | The receiver operating characteristic |
| AUC | the area under the ROC curve |
| FPR | False Positive Rate |
| TPR | True Positive Rate |
| CNA | copy number alteration |
| PSG | protein structure graph |
| GSEA | Gene set enrichment analysis |
| JAK1 | Janus Kinase 1 |
| STAT1 | Signal Transducer And Activator Of Transcription 1 |
| IFIT2 | Interferon Induced Protein With Tetratricopeptide Repeats 2 |
| TCF4 | Transcription Factor 4 |
| MMP10 | Matrix Metallopeptidase 10 |
| SP1 | Sp1 Transcription Factor |
| TME | The tumor microenvironment |
| ECM | extracellular matrix |
| MF | molecular function |
| BP | biological process |
| CC | cellular components |
| EGFR | Epidermal Growth Factor Receptor |
| MAPK | Mitogen-Activated Protein Kinase |
| YAP | Yes Associated Transcriptional Regulator |
| LATS | Large Tumor Suppressor Kinase |
