# Supplementary material for: The LIM Protein AJUBA is a Potential Oncogenic Target and Prognostic Marker in Human Cancer via Pan-Cancer Analysis
Source: Front Cell Dev Biol. 2022 Jul 11;10:921897. doi: 10.3389/fcell.2022.921897 (PMC9309301; doi:10.3389/fcell.2022.921897)
Supplement: Supplementary file 3 [file Image1.pdf]

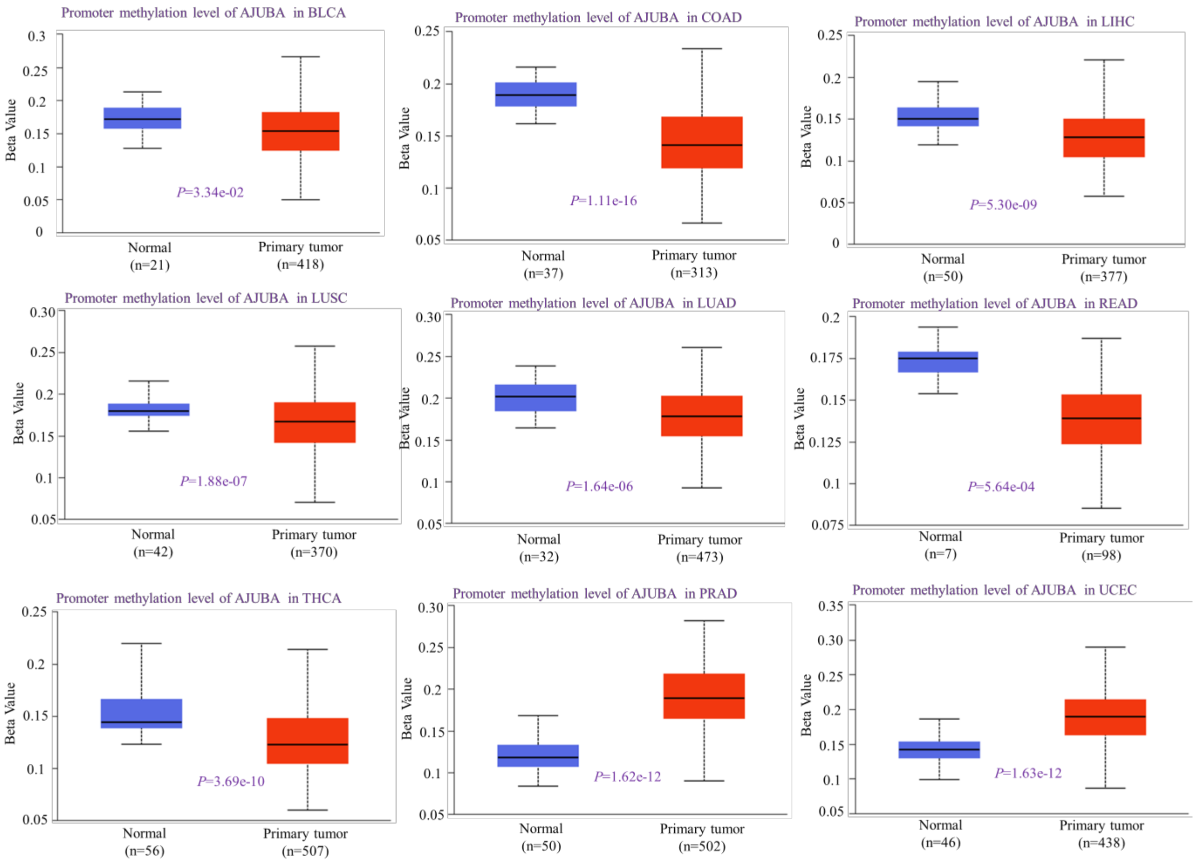

Supplementary Figure 1 AJUBA promoter methylation analysis in different tumors. Methylation levels of AJUBA in different tumors through the UALCAN database (http://ualcan.path.uab.edu/). BLCA, Bladder Urothelial Carcinoma; COAD, Colon adenocarcinoma; LIHC, Liver hepatocellular carcinoma; LUSC, Lung squamous cell carcinoma; LUAD, Lung adenocarcinoma; READ, Rectum adenocarcinoma; THCA, Thyroid carcinoma; PRAD, Prostate adenocarcinoma; UCEC, Uterine Corpus Endometrial Carcinoma.

Supplementary Figure 2

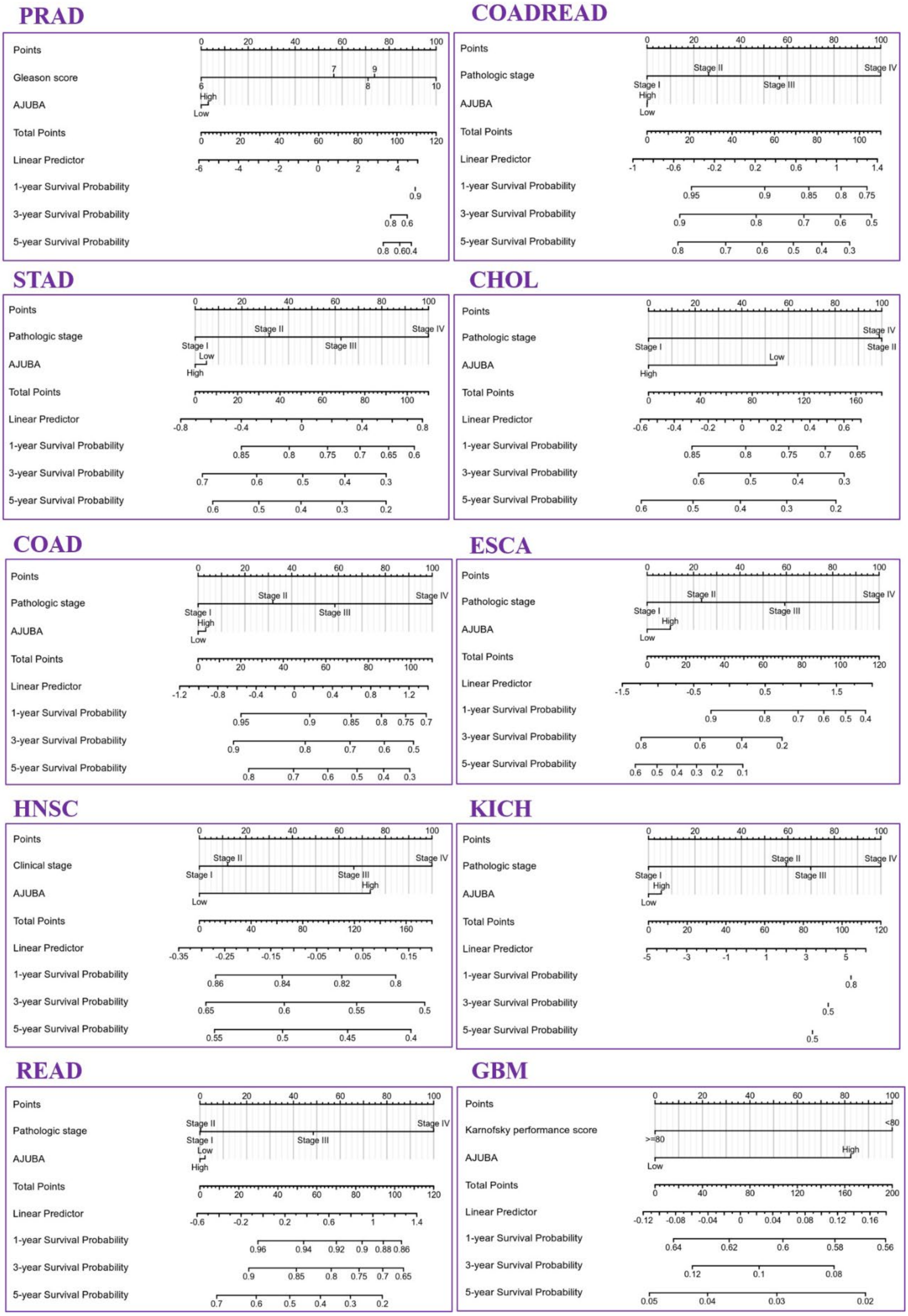

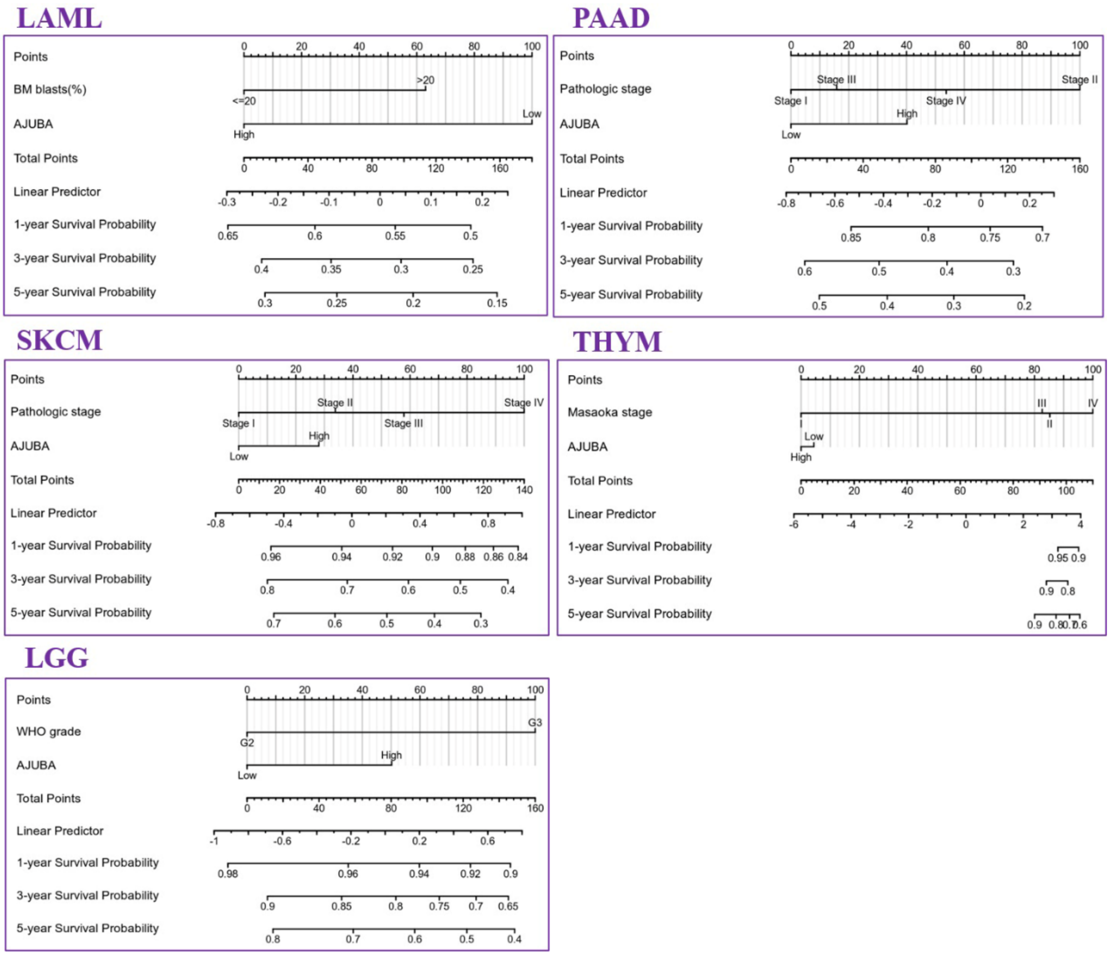

Supplementary Figure 2 Overall survival (OS) predicting with Nomogram analysis at the 1, 3, and 5 years in different tumors. Follow as: PRAD, Prostate adenocarcinoma;COADREAD, Colorectal adenocarcinoma;STAD, Stomach adenocarcinoma;CHOL, Cholangiocarcinoma;COAD, Colon adenocarcinoma;ESCA, Esophageal carcinoma;HNSC, Head and Neck squamous cell carcinoma;KICH, Kidney Chromophobe;READ, Rectum adenocarcinoma;GBM, Glioblastoma multiforme;LAML, Acute Myeloid Leukemia;PAAD, Pancreatic adenocarcinoma;SKCM, Skin Cutaneous Melanoma;THYM, Thymoma;LGG, Lower Grade Glioma.

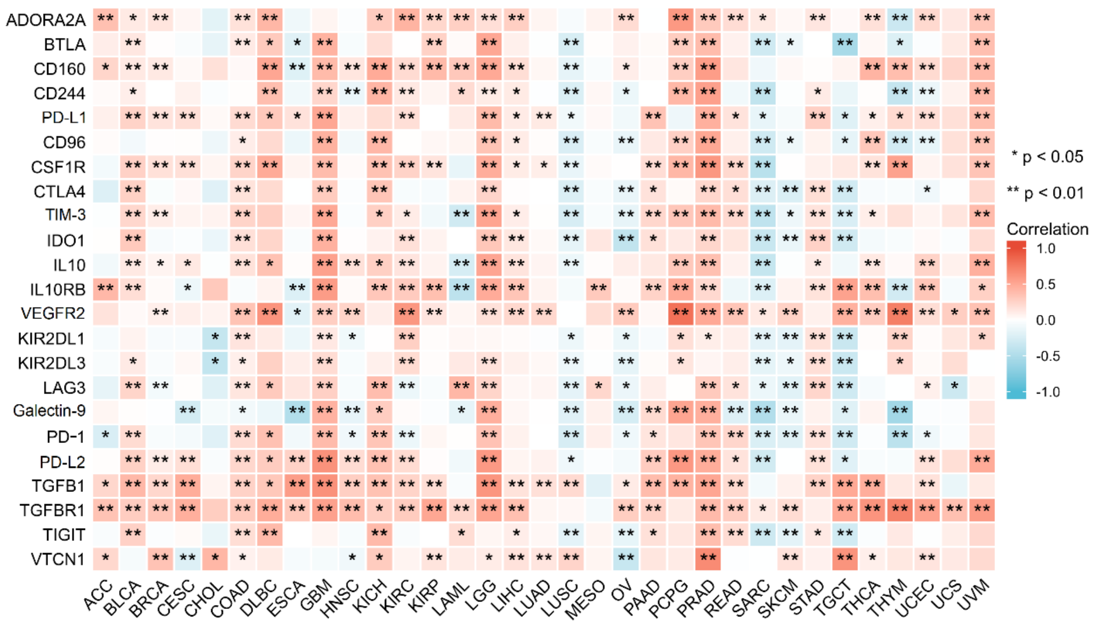

Supplementary Figure 3 Heatmap of the correlation between AJUBA and immunoregulation-related checkpoints in different tumors. *$p < 0.05$, **$p < 0.01$.
